# Determining Pharmacological Mechanisms of Chinese Incompatible Herbs Fuzi and Banxia in Chronic Obstructive Pulmonary Disease: A Systems Pharmacology-Based Study

**DOI:** 10.1155/2020/8365603

**Published:** 2020-12-31

**Authors:** Kaiwen Ni, Xiaolu Cai, Yaling Chen, Linshui Zhou, Ruilin Chen, Suqun Zheng, Zhen Wang

**Affiliations:** ^1^The First Clinical College of Zhejiang Chinese Medical University, Hangzhou, China; ^2^The First Affiliated Hospital of Zhejiang Chinese Medical University, Hangzhou, China

## Abstract

*Aconiti Lateralis Radix Praeparata *(Fuzi) and *Pinelliae Rhizoma* (Banxia) are among the 18 incompatible medications that are forbidden from use in one formulation. However, there is increasing evidence implying that this prohibition is not entirely correct. According to the theory of Chinese traditional medicine, they can be used for the treatment of chronic obstructive pulmonary disease (COPD). Thus, we analyzed the possible approaches for the treatment of COPD using network pharmacology. The active compounds of Fuzi and Banxia (FB) were collected, and their targets were identified. COPD-related targets were obtained by analyzing the differentially expressed genes between COPD patients and healthy individuals, which were expressed using a Venn diagram of COPD and FB. Protein-protein interaction data and network regarding COPD and drugs used were obtained. Gene ontology and Kyoto Encyclopedia of Genes and Genomes pathway analysis were conducted. The gene-pathway network was constructed to screen the key target genes. In total, 34 active compounds and 47 targets of FB were identified; moreover, 7,153 differentially expressed genes were identified between COPD patients and healthy individuals. The functional annotations of target genes were found to be related to mechanisms such as transcription, cytosol, and protein binding; furthermore, 68 pathways including neuroactive ligand-receptor interaction, Kaposi sarcoma-associated herpesvirus infection, apoptosis, and measles were significantly enriched. FOS CASP3, VEGFA, ESR1, and PTGS2 were the core genes in the gene-pathway network of FB for the treatment of COPD. Our results indicated that the effect of FB against COPD may involve the regulation of immunological function through several specific biological processes and their corresponding pathways. This study demonstrates the application of network pharmacology in evaluating mechanisms of action and molecular targets of herb-opponents FB.

## 1. Introduction

As defined by the Global Initiative for Chronic Obstructive Lung Disease (GOLD) Reports 2020, chronic obstructive pulmonary disease (COPD) is characterized by persistent respiratory symptoms and airflow limitation ascribed to airway and/or alveolar abnormalities. Data from the American Lung Association Epidemiology and Statistics Unit show that COPD is projected to be the 3^rd^ by 2020. According to GOLD, smoking cessation is essential in all COPD patients who continue to smoke, whereas bronchodilators, inhaled corticosteroids (ICS), and anti-inflammatory agents are the primary drugs for COPD patients in stable conditions. However, current treatments are only able to slow the progression of COPD [1].

Recently, traditional Chinese medicine (TCM) has been shown to have potential therapeutic properties against COPD [2]. For instance, Bufei Yishen formula exerted its anti-COPD efficacy by restoring Th17/Treg balance via the modulation of STAT3 and activation of STAT5 [3]. Clinically, chronic and progressive dyspnea are the main characteristic symptoms of COPD, and around a third of patients experience cough and sputum production. According to the theory of TCM, the dyspnea may imply depression of the chest yang, while sputum production suggests phlegm stagnant. Additionally, the elderly, the primary population of the disease, are often considered to have declination in the kidney yang. These characteristics mentioned belong to one syndrome called yang-deficiency-with-phlegm-stagnant, in TCM.

In TCM, *Aconiti Lateralis Radix Praeparata* (Fuzi, the dried lateral root of *Aconitum carmichaelii* Debx.) improves COPD's yang deficiency by tonifying fire and enhancing yang. *Pinelliae Rhizoma* (Banxia, the dried tuber of *Pinellia ternata* (Thunb.) Breit.) defends against COPD's phlegm stagnant by drying dampness and resolving phlegm. In addition, it is reported that *P. ternata* can be used to reverse various adverse effects of the withdrawal of ICS, such as a rebound in goblet cell number, mucin 5AC (MUC5AC) expression, and interleukin 1*β* (IL-1*β*) and tumor necrosis factor *α* (TNF-*α*) levels, used for the treatment of COPD [4]. Moreover, patients with COPD have higher levels of systemic inflammation markers [5]. Fuzi has a wide range of biological activities, including anti-inflammatory, antitumor, and immunomodulatory effects, as well as effects on energy metabolism [6].

Cotreatment of FB is not to be used in TCM, as indicated in the *Eighteen Incompatible Medicaments Verse* and first noted in the *Rumen Shiqin* [7], owing to high toxicity and side effects of the combination in one apozem. However, increasing evidence shows that they have no significant harmful effects on humans [8]. One clinical study showed that there was no significant toxicity of cotreatment of FB in the heart, liver, kidney, and blood used in patients with malignant tumors. [9] Moreover, as reported in “*Shanghan Zabing Lun*,” a widely used clinical guidebook of TCM, 2000 years ago, FB were used in a formulation called “*Fuzi-jingmi Tang*.” [10] As for now, we still do some clinical researches using formulations which contained FB. *Xiaoqinglong-Jiafuzi-Decoction*, including both Fuzi and Banxia, significantly reduced COPD patients' modified Medical Research Council (mMRC) scores, and enhanced their lung function. [11] Another clinical research indicated that *Xiaoqinglong-Jiafuzi-Decoction* sharply increased the negative rate of IgE when compared with conventional Western medicine in the treatment of allergic rhinitis. [12] There was no obvious harmful effectiveness of combination of FB mentioned. Thus, we utilized a network pharmacology method to delineate the mechanisms of action and molecular targets of FB for their possible treatment of COPD.

In TCM, multiple herbs are used in complex herbal formulations for various diseases because of their potentially bioactive components that interact with multiple therapeutic targets. The multicomponent, multitarget, and multipathway of formulations means TCM can provide ideal treatments but also comes with great challenges due to the interactions among them [13]. Network pharmacology is a novel approach that attempts to solve these problems, combining systems network analysis with pharmacology. It can be used to illustrate the synergism among compounds and the potential mechanisms of various components and multitarget drugs at the molecular level through the network levels of compound-compound, compound-target, and target-disease interactions. Network pharmacology could promote the understanding of the interactions among compounds, genes, proteins, and diseases; moreover, it is suitable for the study of complex TCM formulations [14]. For example, a feasible system pharmacology model based on chemical, pharmacokinetic, and pharmacological data was developed via a network construction approach to clarify the synergistic mechanisms of Huangqi (*Radix Astragali*) and Huanglian (*Rhizoma Coptidis*) [15]. The active ingredients of Fuzheng Huayu formulation were successfully identified, and the mechanisms by which they inhibit hepatic stellate cell viability were determined using network pharmacology and transcriptomics [16]. In this study, for the first time, we explored the action mechanisms and molecular targets of FB for the treatment of COPD using network pharmacology.

## 2. Materials and Methods

### 2.1. Active Ingredient Screening

The chemical compositions of FB were identified from the Traditional Chinese Medicine Systems Pharmacology Database and Analysis Platform (TCMSP) [17]. Then, we selected candidate compounds with standard oral bioavailability (OB) ≥ 30% and drug-likeness (DL) ≥ 0.18 [18].

A total of 34 identified compounds were imported into the DrugBank database [19] to delineate the relevant targets of FB. According to one relevant literature report, 5 representative alkaloids of Fuzi, aconitine (AC), mesaconitine (MA), benzoylaconine (BAC), benzoylmesaconine (BMA), and benzoylhypaconine (BHA), which were not selected out from the DrugBank were also included in the scope of the study [20]. They are also listed in Table 1.

### 2.2. Identifying COPD-Related Targets

COPD genes were collected from both Genecards and the Online Mendelian Inheritance in Man (OMIM) databases. There was no threshold applied during this step, so as to collect as many genes as possible.

### 2.3. Venn Diagram

We have made a Venn diagram using Venny to show the overlapping genes of COPD and FB.

### 2.4. Network and Protein-Protein Interaction Analysis

Using Cytoscape 3.7.2 software, genes common to the drugs and the disease were connected to visualize the relationship between the compounds used against FB and COPD. Protein-protein interactions (PPI), as per data from STRING, are based on the proteins in common. First, the genes are ranked by calculating the number of times they appear in the network. Then, with the CytoNCA APP, we analyzed degree centrality (DC), closeness centrality (CC), local average connectivity-based method (LAC), betweenness centrality (BC), Eigenvector centrality (EC), and network centrality (NC), all of which indicate a protein's topology potential. It is reported that their definitions and calculation formulas have been used in network pharmacology and system pharmacology [21]. Meanwhile, we counted the string interactins between nodes to figure out the core genes. To make out the core genes, we used CytoHubba to catch top 5 genes ranked by maximal clique centrality (MCC). [22].

### 2.5. Bioinformatic Analysis

#### 2.5.1. Gene Ontology (GO) Analysis

GO analysis, based on the biological processes, cellular components, and molecular functions, places genes that are functionally similar into one group [23]. We used R and Bioconductor [24] to visualize the GO analysis. Functional classifications were analyzed within genes (*p* value cutoff = 0.05, *q* value cutoff = 0.05).

#### 2.5.2. Kyoto Encyclopedia of Genes and Genomes (KEGG) Database

The KEGG database was used to analyze pathways, in the same manner as GO analysis. Significant changes (*p* value cutoff = 0.05, *q* value cutoff = 0.05) were distinguished for further study.

### 2.6. Visualization of Molecular Docking Analysis of Active Components and Core Genes

The protein expressed by core genes and active components were analyzed to figure out the active ingredients which had both minimum binding energy and polar contact. PYMOL was used to visualize docking effect conformation.

## 3. Results

### 3.1. Compound-Target Network Analysis

Twenty-one relevant compounds were obtained from the analysis of Fuzi and 13 from Banxia analysis (Table 1). For Fuzi, the OBs of ignavine, (R)-norcoclaurine, and karanjin are 84.08%, 82.54%, and 69.56%, respectively, which suggests that they may be the most effective active compounds present, while for Banxia, the OBs of (3S, 6S)-3-(benzyl)-6-(4-hydroxybenzyl) piperazine-2, 5-quinone, beta-D-ribofuranoside (xanthine-9), and stigmasterol are 46.89%, 44.72%, and 43.83%, respectively.

All 39 compounds were imported into the DrugBank database to further delineate the targets of FB. After removing 23 compounds with no known corresponding targets, 6 active compounds in Fuzi and 10 in Banxia were identified. From the active compounds, all 47 targets were identified, 13 for Fuzi (Table S1) and 41 for Banxia (Table S2). Genetic symbols of all targets are included for clarity (Table 1). Additionally, we have listed the number of targets corresponding to different components in FB (Table S3) and included the gene symbols of the targets for clarity. Unfortunately, the 5 compounds that were added were filtered without knowing the corresponding target genes.

### 3.2. Identifying COPD-Related Targets

COPD genes were collected from both Genecards and the OMIM databases, ultimately resulting in a collection of 7,153 related targets.

### 3.3. Venn Diagram

**Figure 1:**
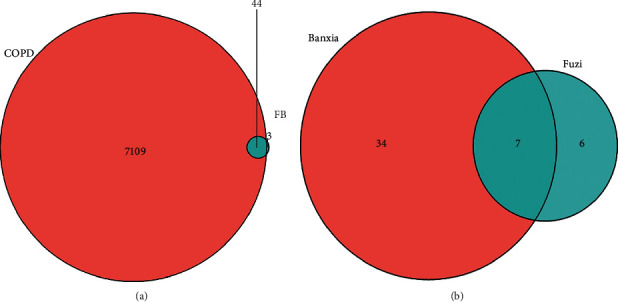
(a) Venn diagram of overlapping genes related to COPD and FB. Blue indicates genes related to FB, and pink indicates genes related to COPD. (b) Venn diagram of overlapping genes related to Fuzi and Banxia. Blue indicates genes related to Fuzi, and pink indicates genes related to Banxia.

A Venn diagram (Figure 1(a)) was used to depict the overlapping genes of COPD and FB. The analysis showed that 44 genes are related to both COPD and FB. Another Venn diagram (Figure 1(b)) was used depict the 7 common genes of FB. The 2 drugs may work synergistically through these common genes: ESR1, PTGS1, PRSS1, PGR, NCOA1, NCOA2, and NR3C2.

### 3.4. Network Analysis

**Figure 2:**
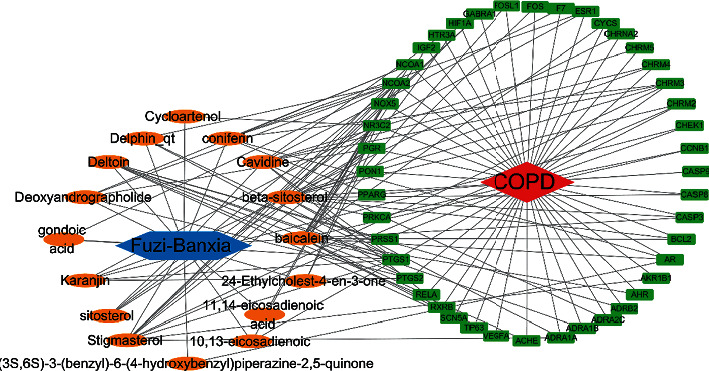
Network analysis of common targets for COPD and FB. Red represents COPD, green represents relevant genes, orange represents the ingredients, and blue represents the impairing drugs.

Using Cytoscape 3.7.2 software, the 44 common genes, alongside the network between FB and COPD, were set up and visualized (Figure 2). Between the 16 compounds in both FB and the 44 genes with overlapping relationships with COPD, 147 edges were analyzed. These edges indicate the compound-target interactions.

### 3.5. PPI Analysis

**Figure 3:**
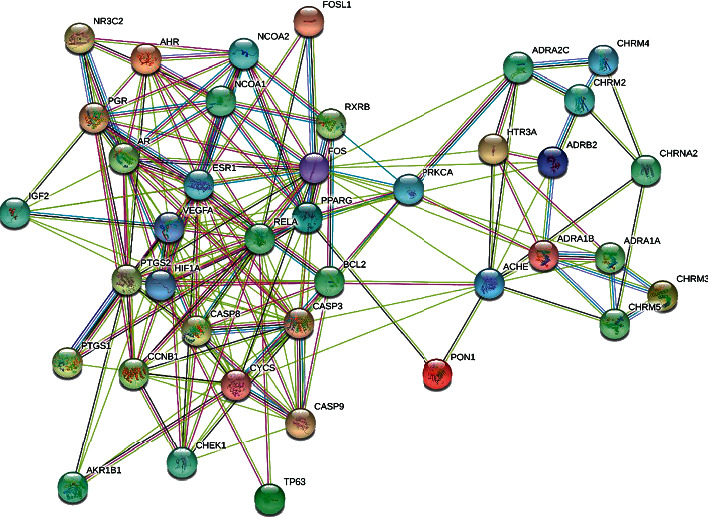
PPI network for COPD and FB.

**Figure 4:**
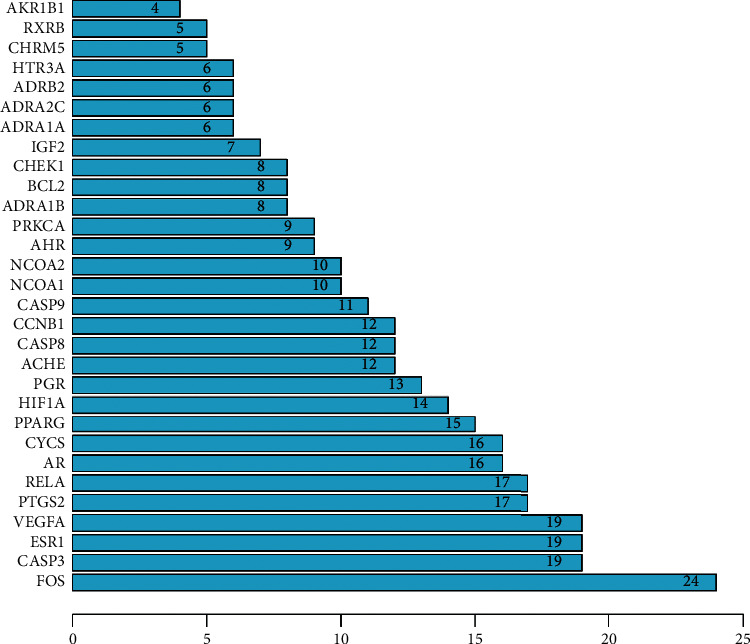
Bar plot of PPI for COPD and FB. The number showed the appearance times of the genes in PPI. Larger number means more important.

Based on the same 44 targets, after removal of discrete points, a PPI network was generated (Figure 3), which included 39 nodes and 186 edges, representing 39 interacting proteins and 186 interactions. We counted the appearance of genes in the PPI network and ranked their value roughly (Figure 4).

**Figure 5:**
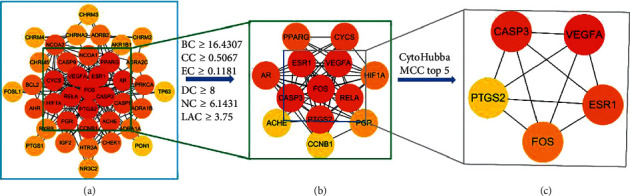
(a). Identification of targets of FB against COPD. From red to yellow indicates higher DC scores to low DC scores. (b) Targets identified above the medians. From red to yellow indicates higher DC scores to lower DC scores. (c). Core genes of FB and COPD.

With the use of the CytoNCA APP, we calculated the median of BC, CC, EC, DC, NC, and LAC as 16.4307, 0.5067, 0.1181, 8, 6.1431, and 3.75, respectively. We selected the targets that demonstrated higher indices for each median, for further analysis. Eventually, 13 targets were obtained: FOS, CASP3, VEGFA, ESR1, PTGS2, RELA, AR, CYCS, HIF1A, PGR, PPARG, NCOA1, and NCOA2. In way of CytoHubba, ranked by MCC, we got top 5 genes as core genes- FOS, CASP3, VEGFA, ESR1, and PTGS2 (Table 2 and Figure 5).

### 3.6. Molecular Docking Analysis

**Figure 6:**
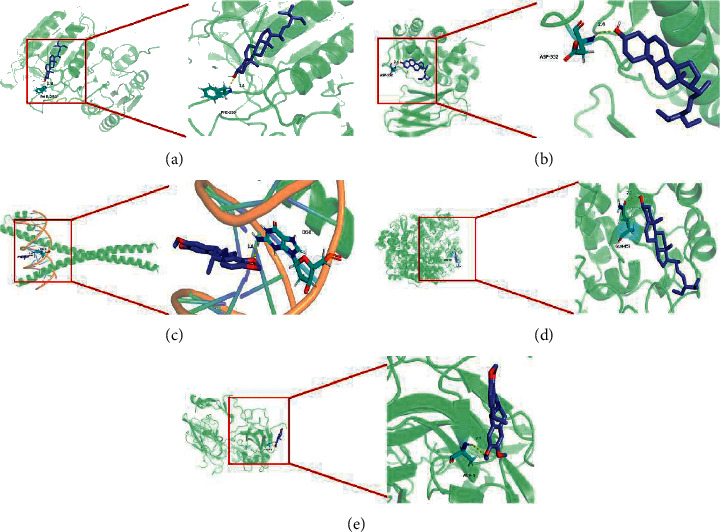
Molecular docking charts of core genes and active components. (a) CASP3 and Stigmasterol. (b) ESR1 and Stigmasterol. (c) FOS and Cavidine. (d) PTGS2 and Stigmasterol. (e) VEGFA and Cavidine.

Molecular docking of the active components and core genes with high mutual attraction is listed in Table 3, while the relating images are shown in Figure 6.

#### 3.6.1. GO

**Figure 7:**
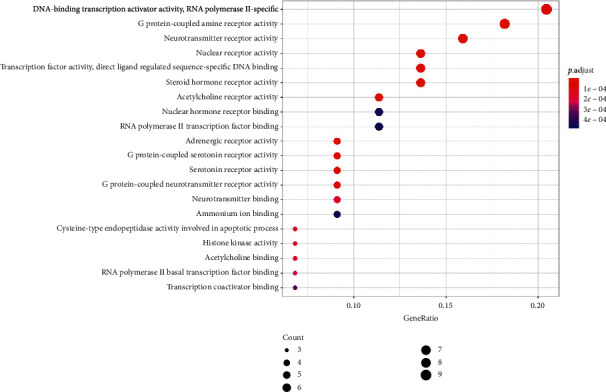
Gene ontology terms of candidate targets of FB against COPD. They were identified by the threshold of *p* value cutoff ≤0.05. The size of the spot represents the number of genes, and the color represents the adjusted *p* value.

The 13 identified candidate targets were analyzed using Bioconducter by R (threshold; *p* value cutoff = 0.05, *q* value cutoff = 0.05). The data of GO analysis are listed in Table S4. Twenty GO terms were analyzed (Figure 7).

#### 3.6.2. KEGG

**Figure 8:**
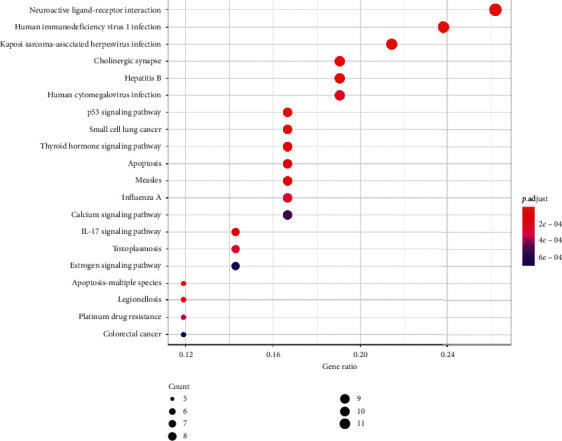
KEGG pathway identification of FB candidate targets against COPD. They were identified by the threshold of value cutoff ≤0.05. The size of the spot represents the number of genes, and the color represents the adjusted value.

**Figure 9:**
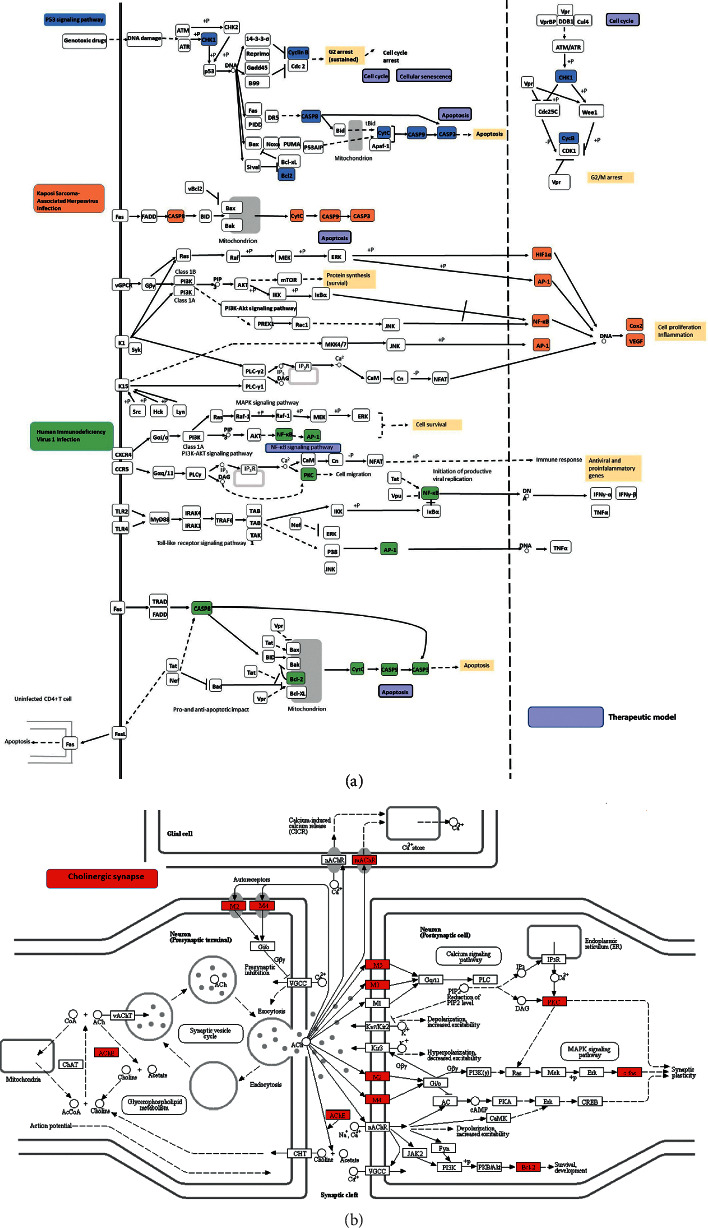
(a) KEGG pathway details identified by FB against COPD. Three pathways (shown in different colors) consisted the compressed KEGG pathway. The solid and dashed arrows mean direct and indirect activations, and the T arrows indicate the inhibition effects. (b) Cholinergic synapse details identified by FB against COPD.

The critical pathways involved in the FB treatment of COPD were identified by KEGG pathway analysis (Figure 8). The data of KEGG analysis are shown in Table S5. There were 68 enhanced pathways identified (*p* value cutoff = 0.05, *q* value cutoff = 0.05), including neuroactive ligand-receptor interaction, human immunodeficiency virus 1 infection, Kaposi sarcoma-associated herpesvirus infection, cholinergic synapse, and hepatitis B.  Figure 9 shows the details of four pathways through which the genes were primarily distributed in the p53 signaling pathway, apoptosis pathway, and cholinergic synapse pathway.

**Figure 10:**
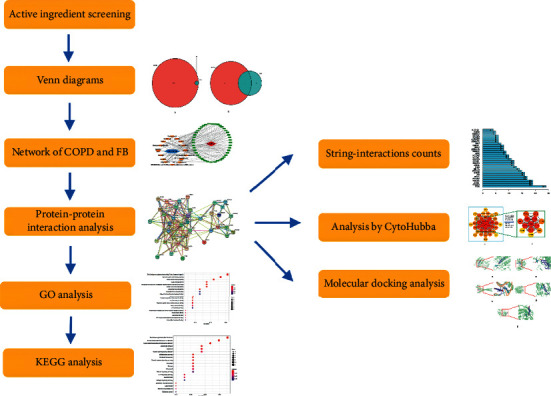
Article flow chart.

The article flow chart to show our job is given in Figure 10.

## 4. Discussion

Network-based methods are expected to make drug discovery breakthroughs by increasing our understanding of drug actions using multiple layers of information. Moreover, it provides additional support in the development of drug design and determination of the mechanism of action [25]. It is also a useful tool to elucidate the potential mechanisms of action of Chinese herbs on diseases, which may determine whether some theories of TCM are correct or incorrect.

As mentioned above, FB may treat COPD; thus, we evaluated this possibility using network pharmacology. Previously, FB were thought to be incompatible based on the commonsense logic employed in TCM. However, so far, no significant and definite adverse effects have been reported by administering a combination of FB. Many studies have focused on the side effects (hepatorenal toxicity) of FB, and a few articles focus on the therapeutic effect of them. We think that it is better to discuss the side effects of drugs in the context of sufficient clinical efficacy. Drugs possess therapeutic effects and may also cause complications; for example, tacrolimus (FK506) and other immunosuppressants inhibit the immunity of patients undergoing transplantation, making them vulnerable to infectious diseases (e.g., BK virus infection) or hypertension mentioned in the drug specification. However, owing to their therapeutic effect, their risks are tolerated. Therefore, it is necessary to study and overcome the possible side effects of FB; however, proving the drugs' therapeutic effect seems to have more clinical value.

In this study, we evaluated some valuable compounds, such as baicalein in Banxia and ignavine and karanjin in Fuzi. Ignavine is a novel allosteric modulator of the *μ* opioid receptor and has an analgesic effect in vivo [26]. Karanjin decreases reactive oxygen species (ROS) levels by inhibiting B-cells inhibitor (I‐*κ*B), resulting in the restriction of nuclear factor kappa-light-chain-enhancer of activated B-cells (NF‐*κ*B) nuclear translocation. It can also reduce DNA damage by increasing p53 expression [27]. Karanjin has been shown to reduce TNF-*α* production and to have a potent inhibitory effect on nitric oxide and reactive oxygen species production [28]. As oxidative stress is a major contributor to the pathogenesis of COPD, karanjin may be effective in the treatment of COPD. Although AC, HA, MA, BAC, BHA, and BMA were filtered out, they are the main research targets in experiments related to Fuzi. A study based on ultra-high-performance liquid chromatography coupled tandem mass spectrometry (UPLC-MS/MS) showed that the parent compounds (AC, HA, and MA), which are more toxic than their corresponding secondary metabolites (BAC, BHA, and BMA), would be eliminated more rapidly [29]. In turn, the components found in this study, which are not regarded as the key active components of Fuzi, may have new and significant properties, which may be potentially demonstrated in subsequent studies. Baicalein, from Banxia, has been shown to relax rat tracheal smooth muscle, as effectively as theophylline [30].

This study demonstrates that there are 44 overlapping genes between FB and COPD (Figure 1). The PPI network analysis of FB's putative targets and COPD-related targets was explored and identified common targets that scored higher than the medians in the analyzed parameters. The top 13 targets identified were FOS, CASP3, VEGFA, ESR1, PTGS2, RELA, AR, CYCS, HIF1A, PGR, PPARG, NCOA1, and NCOA2.

Oxidative stress can destroy biomacromolecules in tissues, leading to cell dysfunction or cell death [31]. This study shows that the correlated target genes or pathways are highly associated with oxidative stress, which is a major contributor to the pathogenesis of COPD. For example, Recuperating Lung Decoction could inhibit the MAPK/AP-1 signaling pathway to downregulate oxidative stress, resulting in the improvement of antioxidation of COPD. [32] Indeed, apoptosis was the second most indicated pathway in KEGG analysis. Whether in p53 signaling way, Kaposi sarcoma-associated herpesvirus infection pathway, or human immunodeficiency virus 1 infection pathway, the genes were primarily involved in apoptosis pathway (Figure 10). CSE induced endothelial apoptosis in COPD through the ERK pathway [33]. In turn, oxidative stress can promote the development of lung inflammation and form a chronic state of mucus secretion. The Fos-related antigen-1 (Fra-1) transcription factor is thought to play a key role in promoting chronic cigarette smoke- (CS-) induced lung macrophagic inflammation in vivo and experiments have shown that the absence of Fra-1 in mice bone marrow can reduce the expression of inflammatory factors and the aggregation of macrophages in the lung [34]. Additionally, Fos-related antigen-2 (Fra-2) expression has also been described in COPD for the upregulation of monocyte-derived macrophages upon CS stimulation [35]. Activating transcription factor 3 (ATF3) may be involved in transcriptional promotion of CS-induced MUC5AC expression, which is a key pathologic feature of COPD in airway epithelial cells. Moreover, the knockout of ATF3 can significantly reduce the production of mucus and reduce the chronic mucus hypersecretion of airways [36].

Protease-antiprotease imbalance is the third main pathogenic mechanism of COPD. CS leads to the infiltration of many neutrophils and macrophages in the lung, releasing excessive neutrophil elastase and matrix metalloproteinase (MMP). Through the protease antiprotein imbalance mechanism, the expression of placenta growth factor (PGF) is increased and drives caspase-3- and caspase-9-dependent apoptosis in bronchial epithelial cells [37]. Pulmonary emphysema, a significant characteristic of COPD, is considered to result from the epithelial cell death caused by smoking [38]. It shows that the normal pulling force of alveoli to small airways has been reduced, making the alveoli easily collapsible with obviously reduced elasticity. The failure of the small airway is an important but easily overlooked mechanism of COPD because it occurs in the very early stages of the disease, and continues throughout the process [39]. Small airway disease is composed of small airway inflammation, fibrotic tissue, and lumen mucus plug. The the abovementioned mechanisms jointly promote the most characteristic symptom of COPD: continuous airflow restriction. ICS are very effective in the treatment of COPD. However, ICS resistance is becoming a major barrier to the effective treatment of COPD [40]. There has been evidence indicating that the loss of Hsp90 could contribute to steroid resistance in COPD [41]. Meanwhile, one clinical study has illustrated that smokers with COPD possessed significantly increased expression levels of p53 when compared to smokers without COPD and normal subjects, and at the same time, increased cleaved caspase-3 may also promote apoptosis [42]. All these biological processes were analyzed in the present study. As shown in Figure 8, the caspase-3, caspase-9, and p53 signaling pathways form the core of the network between FB and COPD. Moreover, the highlighted parts in the major pathways are mainly related to apoptosis.

As is demonstrated in the present study, FB are multicomponent, multitarget, and multipathway drugs. A total of 68 pathways were enhanced in this drug, including those related to IL-17 signaling, P53 signaling, cholinergic synapse, and apoptosis. IL-17A regulates airway inflammation, oxidative stress, and the reduction of steroid sensitivity [43], as well as attenuating IFN-*λ* expression [44] in COPD. Cellular senescence and apoptosis of alveolar epithelial cells in lung tissues are important characteristics of COPD pathogenesis. p53 is a tumor suppressor, which can induce cellular senescence of type II alveolar epithelial cells (AECII) [45]. p53 expression in the cytoplasm also increased with CSE treatment. In addition, the interaction of p53 with Parkin (a core mitophagy-regulating protein) was highly increased during CSE-induced cellular senescence [46]. The cholinergic anti-inflammatory pathway was involved in regulating inflammation; moreover, COPD mouse showed high levels of AChE and nicotinic acetylcholine receptor *α*7 subunit (*α*7nAChR) [47]. The nonneuronal cholinergic system was overexpressed in neutrophils of COPD patients, such as the increase of muscarinic receptors (M2, M4, and M5) expression [48]. Additionally, in this study, several other viral pathways were enhanced. Studies which use polymerase chain reaction have demonstrated the presence of viruses in 80–85% of asthma exacerbations in children and 60–80% of exacerbations in adults, after viral clearance, and symptom resolution follows, but lasting inflammatory markers and oxidative stress remain [49], which suggests a perpetual autoimmune response. FB may regulate the immunological function through some specific infections' pathways, such as Kaposi sarcoma-associated herpesvirus infection, human immunodeficiency virus 1 infection, human cytomegalovirus infection, hepatitis C, influenza A, and Epstein–Barr virus infections. COPD is an independent risk factor for non-small-cell lung cancer (NSCLC) and significantly associated with lung cancer mortality [50]. Fuzi reduced the proportion of Treg cells, decreased serum levels of cytokines and transforming growth factor (TGF)-*β*, and downregulated the expression of programmed death ligand-1 in mice, which suggests that Fuzi has immunomodulation properties to improve radiotherapy against lung cancer [51]. In this study, the pathways mentioned above were enhanced as well.

In the network we set up, FOS may be the core target gene. In addition, CASP3 and VEGFA may be key target genes. The overexpression of FOS and CASP3 suggests excessive apoptosis [52]. Additionally, activated c-Fos can influence colon cancer invasion [53]; c-Fos in T24 cells can induce significant cell morphology changes, reduce viability, and increase cell death [54]. In osteosarcoma (OS), only tumors expressing both epidermal growth factor receptor (EGFR) and c-Fos respond to anti-EGFR therapy [55]. CASP3 plays a central role in executing cell apoptosis; carcinogenesis studies have found that CASP3 polymorphisms and smoking interactions were related to a higher risk of lung cancer [56], whereas lower expression of CASP3 is linked to a higher risk of NSCLC [57, 58]. Although no difference in the expression of ESR1 was observed between patients and healthy individuals, the increased expression of enzymes involved in the local synthesis of active estrogens were observed [59]. Vascular endothelial-derived growth factor (VEGF) may play a key role in ongoing vascular remodeling processes, a key characteristic sign of COPD, in the distal lung compartments [60].

The mechanisms of action and molecular targets of FB against COPD were explored using a network pharmacology in this study. FB regulates some biological processes, such as gene expression, apoptotic processes, neuroactive ligand-receptor interaction and p53 class mediators, and cholinergic synapse, and the enhanced pathways include the IL-17 signaling and thyroid hormone signaling pathway. FB may regulate immunological function through some specific viral pathways, such as Kaposi sarcoma-associated herpesvirus infection, human immunodeficiency virus 1 infection, human cytomegalovirus infection, hepatitis C and influenza A, and Epstein–Barr virus infection, which are associated with COPD. As judged by DC, the top 3 key genes are FOS, CASP3, and ESR1 in the PPI of FB for the treatment of COPD. Network pharmacology seems to be a suitable approach for the study of complex TCM incompatible drugs; however, it may filter compounds that may be associated with unknown target genes or lower OB and DL, such as BAC, BHA, and BMA identified in this study. Moreover, various formulas with effective compositions may be produced using the decocting process, instead of the original preparation methods; the compounds so obtained may have different molecular structures because they are obtained by boiling. As TCM research is difficult, although not an ideal approach, network pharmacology does offer a new and reliable approach to analyze TCMs and associate them with diseases. It can be used to verify whether a theory of TCM is valid through modern experimental methods in order to conveniently and accurately explain how drugs function.

## Figures and Tables

**Table 1:** Components of Fuzi and Banxia and their parameters.

Id	Name	OB	DL	Source
MOL002421	Ignavine	84.08	0.25	Fuzi
MOL002419	(R)-norcoclaurine	82.54	0.21	Fuzi
MOL002398	karanjin	69.56	0.34	Fuzi
MOL002388	Delphin_qt	57.76	0.28	Fuzi
MOL002395	Deoxyandrographolide	56.3	0.31	Fuzi
MOL002415	6-Demethyldesoline	51.87	0.66	Fuzi
MOL002397	Karakoline	51.73	0.73	Fuzi
MOL002422	Isotalatizidine	50.82	0.73	Fuzi
MOL002392	Deltoin	46.69	0.37	Fuzi
MOL002401	Neokadsuranic acid B	43.1	0.85	Fuzi
MOL002433	(3R, 8S, 9R, 10R, 13R, 14S, 17R)-3-hydroxy-4, 4, 9, 13, 14-pentamethyl-17-[(E, 2R)-6-methyl-7-[(2R, 3R, 4S, 5S, 6R)-3, 4, 5-trihydroxy-6-[[(2R, 3R, 4S, 5S, 6R)-3, 4, 5-trihydroxy-6-(hydroxymethyl)oxan-2-yl]oxymethyl]oxan-2-yl]oxyhept-5-en-2-yl]-1, 2, 3, 7, 8, 10, 12, 15, 16, 17-decahydr	41.52	0.22	Fuzi
MOL002211	11, 14-Eicosadienoic acid	39.99	0.2	Fuzi
MOL002406	2, 7-Dideacetyl-2, 7-dibenzoyl-taxayunnanine F	39.43	0.38	Fuzi
MOL002434	Carnosifloside I_qt	38.16	0.8	Fuzi
MOL000359	Sitosterol	36.91	0.75	Fuzi
MOL002393	Demethyldelavaine A	34.52	0.18	Fuzi
MOL002394	Demethyldelavaine B	34.52	0.18	Fuzi
MOL002410	Benzoylnapelline	34.06	0.53	Fuzi
MOL002423	Jesaconitine	33.41	0.19	Fuzi
MOL000538	Hypaconitine	31.39	0.26	Fuzi
MOL002416	Deoxyaconitine	30.96	0.24	Fuzi
MOL002089	Mesaconitine	8.7	0.25	Fuzi
MOL002424	Aconitine	7.87	0.23	Fuzi
MOL002408	Benzoylaconine	12.83	0.25	Fuzi
MOL002409	Benzoylhypaconine	8.7	0.29	Fuzi
MOL002093	Benzoylmesaconine	8.55	0.27	Fuzi
MOL006957	(3S, 6S)-3-(benzyl)-6-(4-hydroxybenzyl) piperazine-2, 5-quinone	46.89	0.27	Banxia
MOL006967	beta-D-ribofuranoside, xanthine-9	44.72	0.21	Banxia
MOL000449	Stigmasterol	43.83	0.76	Banxia
MOL006937	12, 13-Epoxy-9-hydroxynonadeca-7, 10-dienoic acid	42.15	0.24	Banxia
MOL002776	Baicalin	40.12	0.75	Banxia
MOL006936	10, 13-Eicosadienoic	39.99	0.2	Banxia
MOL003578	Cycloartenol	38.69	0.78	Banxia
MOL000358	Beta-sitosterol	36.91	0.75	Banxia
MOL001755	24-Ethylcholest-4-en-3-one	36.08	0.76	Banxia
MOL002670	Cavidine	35.64	0.81	Banxia
MOL002714	Baicalein	33.52	0.21	Banxia
MOL000519	Coniferin	31.11	0.32	Banxia
MOL005030	Gondoic acid	30.7	0.2	Banxia

**Table 2:** Targets identified above each median.

	Degree	Eigenvector	LAC	Betweenness	Closeness	Network
FOS	24	0.30	8.58	379.38	0.73	19.12
CASP3	19	0.28	9.68	75.48	0.63	15.54
VEGFA	19	0.28	9.68	56.57	0.61	16.07
ESR1	19	0.28	9.79	52.61	0.60	16.30
PTGS2	17	0.26	9.18	65.09	0.61	13.13
RELA	17	0.25	8.47	70.93	0.59	13.15
AR	16	0.25	9.25	38.73	0.58	13.01
CYCS	16	0.24	8.25	62.59	0.60	12.18
HIF1A	14	0.22	7.29	45.81	0.57	9.76
PGR	13	0.20	7.69	26.13	0.55	10.18
PPARG	15	0.19	5.47	108.42	0.58	8.28
NCOA1	10	0.14	5.40	22.35	0.53	7.01
NCOA2	10	0.14	5.40	22.35	0.53	7.01

**Table 3:** Molecular docking of core genes and active components.

Core gene	Active component	Binding energy
Caspase-3	Stigmasterol	−5.35
ESR1	Stigmasterol	−5.41
FOS	Cavidine	−5.38
PTGS2	Stigmasterol	−4.01
VEGFA	Cavidine	−4.07

## Data Availability

The data used to support the findings of this study are included within the supplementary material files.

## Supplementary Materials

Supplementary Materials Supplementary Table 1. Gene targets of Fuzi. Supplementary Table 2. Gene targets of Banxia. Supplementary Table 3. Number of targets corresponding to different components. Supplementary Table 4. The data of GO enrichment analysis. Supplementary Table 5. The data of KEGG enrichment analysis.
