# Supplementary material for: Determining Pharmacological Mechanisms of Chinese Incompatible Herbs Fuzi and Banxia in Chronic Obstructive Pulmonary Disease: A Systems Pharmacology-Based Study
Source: Evid Based Complement Alternat Med. 2020 Dec 31;2020:8365603. doi: 10.1155/2020/8365603 (PMC7790578; doi:10.1155/2020/8365603)
Supplement: Supplementary Materials — Supplementary Table 1. Gene targets of Fuzi. Supplementary Table 2. Gene targets of Banxia. Supplementary Table 3. Number of targets corresponding to different components. Supplementary Table 4. The data of GO enrichment analysis. Supplementary Table 5. The data of KEGG enrichment analysis. [file 8365603.f1.zip › 8365603.f1/GO..pdf]

ID	Description
GO:0033613	activating transcription factor binding
GO:0046982	protein heterodimerization activity
GO:0042805	actinin binding
GO:0004879	nuclear receptor activity
GO:0098531	transcription factor activity, direct ligand regulated sequence-specific DNA binding
GO:0003707	steroid hormone receptor activity
GO:0001228	DNA-binding transcription activator activity, RNA polymerase II-specific
GO:0000978	RNA polymerase II proximal promoter sequence-specific DNA binding
GO:0003682	chromatin binding
GO:0000987	proximal promoter sequence-specific DNA binding
GO:0005126	cytokine receptor binding
GO:0005172	vascular endothelial growth factor receptor binding
GO:0016004	phospholipase activator activity
GO:0004861	cyclin-dependent protein serine/threonine kinase inhibitor activity
GO:0004953	icosanoid receptor activity
GO:0042301	phosphate ion binding
GO:0060229	lipase activator activity
GO:0005161	platelet-derived growth factor receptor binding
GO:0036041	long-chain fatty acid binding
GO:0046965	retinoid X receptor binding
GO:0005123	death receptor binding
GO:0016538	cyclin-dependent protein serine/threonine kinase regulator activity
GO:0004190	aspartic-type endopeptidase activity
GO:0000979	RNA polymerase II core promoter sequence-specific DNA binding
GO:0001223	transcription coactivator binding
GO:0051393	alpha-actinin binding
GO:0070001	aspartic-type peptidase activity
GO:0070412	R-SMAD binding
GO:1901567	fatty acid derivative binding
GO:0001968	fibronectin binding
GO:0042974	retinoic acid receptor binding
GO:0005504	fatty acid binding
GO:0042056	chemoattractant activity
GO:0051059	NF-kappaB binding
GO:0030291	protein serine/threonine kinase inhibitor activity
GO:0001046	core promoter sequence-specific DNA binding
GO:0001102	RNA polymerase II activating transcription factor binding
GO:0001221	transcription cofactor binding
GO:0030331	estrogen receptor binding
GO:0001047	core promoter binding
GO:0050840	extracellular matrix binding
GO:0030374	nuclear receptor transcription coactivator activity
GO:0032813	tumor necrosis factor receptor superfamily binding
GO:0004722	protein serine/threonine phosphatase activity
GO:0070888	E-box binding
GO:0004197	cysteine-type endopeptidase activity
GO:0043621	protein self-association
GO:0033293	monocarboxylic acid binding
GO:0070491	repressing transcription factor binding
GO:0004860	protein kinase inhibitor activity

GeneRatio	BgRatio	pvalue	p.adjust	qvalue	geneID	Count
2020/3/7	64/16969	1.77E-06	1.74E-04	5.78E-05	2353/5468/5970	3
2020/4/7	497/16969	2.37E-05	1.16E-03	3.87E-04	2353/5468/5970/7422	4
2020/2/7	29/16969	5.89E-05	1.92E-03	6.41E-04	5468/5970	2
2020/2/7	46/16969	1.50E-04	2.93E-03	9.77E-04	5468/5241	2
2020/2/7	46/16969	1.50E-04	2.93E-03	9.77E-04	5468/5241	2
2020/2/7	54/16969	2.07E-04	3.37E-03	1.12E-03	5468/5241	2
2020/3/7	390/16969	3.94E-04	5.51E-03	1.84E-03	2353/5970/5241	3
2020/3/7	432/16969	5.31E-04	5.95E-03	1.98E-03	5468/5970/5241	3
2020/3/7	437/16969	5.50E-04	5.95E-03	1.98E-03	2353/5468/5970	3
2020/3/7	452/16969	6.07E-04	5.95E-03	1.98E-03	5468/5970/5241	3
2020/2/7	210/16969	3.07E-03	2.74E-02	9.12E-03	836/7422	2
2020/1/7	11/16969	4.53E-03	2.97E-02	9.90E-03	7422	1
2020/1/7	11/16969	4.53E-03	2.97E-02	9.90E-03	836	1
2020/1/7	12/16969	4.94E-03	2.97E-02	9.90E-03	836	1
2020/1/7	12/16969	4.94E-03	2.97E-02	9.90E-03	5468	1
2020/1/7	12/16969	4.94E-03	2.97E-02	9.90E-03	5970	1
2020/1/7	13/16969	5.35E-03	2.97E-02	9.90E-03	836	1
2020/1/7	14/16969	5.76E-03	2.97E-02	9.90E-03	7422	1
2020/1/7	14/16969	5.76E-03	2.97E-02	9.90E-03	5468	1
2020/1/7	15/16969	6.17E-03	3.02E-02	1.01E-02	5468	1
2020/1/7	17/16969	6.99E-03	3.12E-02	1.04E-02	836	1
2020/1/7	18/16969	7.40E-03	3.12E-02	1.04E-02	836	1
2020/1/7	20/16969	8.22E-03	3.12E-02	1.04E-02	836	1
2020/1/7	21/16969	8.63E-03	3.12E-02	1.04E-02	2353	1
2020/1/7	21/16969	8.63E-03	3.12E-02	1.04E-02	5970	1
2020/1/7	21/16969	8.63E-03	3.12E-02	1.04E-02	5468	1
2020/1/7	22/16969	9.04E-03	3.12E-02	1.04E-02	836	1
2020/1/7	23/16969	9.45E-03	3.12E-02	1.04E-02	2353	1
2020/1/7	23/16969	9.45E-03	3.12E-02	1.04E-02	5468	1
2020/1/7	24/16969	9.86E-03	3.12E-02	1.04E-02	7422	1
2020/1/7	24/16969	9.86E-03	3.12E-02	1.04E-02	5468	1
2020/1/7	28/16969	1.15E-02	3.43E-02	1.14E-02	5468	1
2020/1/7	29/16969	1.19E-02	3.43E-02	1.14E-02	7422	1
2020/1/7	29/16969	1.19E-02	3.43E-02	1.14E-02	5970	1
2020/1/7	30/16969	1.23E-02	3.45E-02	1.15E-02	836	1
2020/1/7	32/16969	1.31E-02	3.57E-02	1.19E-02	2353	1
2020/1/7	37/16969	1.52E-02	4.02E-02	1.34E-02	2353	1
2020/1/7	38/16969	1.56E-02	4.02E-02	1.34E-02	5970	1
2020/1/7	41/16969	1.68E-02	4.20E-02	1.40E-02	5468	1
2020/1/7	42/16969	1.72E-02	4.20E-02	1.40E-02	2353	1
2020/1/7	43/16969	1.76E-02	4.20E-02	1.40E-02	7422	1
2020/1/7	45/16969	1.84E-02	4.20E-02	1.40E-02	5468	1
2020/1/7	45/16969	1.84E-02	4.20E-02	1.40E-02	836	1
2020/1/7	49/16969	2.00E-02	4.36E-02	1.45E-02	54205	1
2020/1/7	49/16969	2.00E-02	4.36E-02	1.45E-02	5468	1
2020/1/7	56/16969	2.29E-02	4.77E-02	1.59E-02	836	1
2020/1/7	56/16969	2.29E-02	4.77E-02	1.59E-02	5468	1
2020/1/7	59/16969	2.41E-02	4.80E-02	1.60E-02	5468	1
2020/1/7	59/16969	2.41E-02	4.80E-02	1.60E-02	5970	1
2020/1/7	60/16969	2.45E-02	4.80E-02	1.60E-02	836	1
