# Supplementary material for: Determining Pharmacological Mechanisms of Chinese Incompatible Herbs Fuzi and Banxia in Chronic Obstructive Pulmonary Disease: A Systems Pharmacology-Based Study
Source: Evid Based Complement Alternat Med. 2020 Dec 31;2020:8365603. doi: 10.1155/2020/8365603 (PMC7790578; doi:10.1155/2020/8365603)
Supplement: Supplementary Materials — Supplementary Table 1. Gene targets of Fuzi. Supplementary Table 2. Gene targets of Banxia. Supplementary Table 3. Number of targets corresponding to different components. Supplementary Table 4. The data of GO enrichment analysis. Supplementary Table 5. The data of KEGG enrichment analysis. [file 8365603.f1.zip › 8365603.f1/KEGG..pdf]

ID	Description	GeneRatio	BgRatio
hsa05167	Kaposi sarcoma-associated herpesvirus infection	2020/5/7	186/7946
hsa04210	Apoptosis	2020/4/7	136/7946
hsa05162	Measles	2020/4/7	138/7946
hsa05161	Hepatitis B	2020/4/7	162/7946
hsa05134	Legionellosis	2020/3/7	57/7946
hsa05130	Pathogenic Escherichia coli infection	2020/4/7	202/7946
hsa05170	Human immunodeficiency virus 1 infection	2020/4/7	212/7946
hsa05163	Human cytomegalovirus infection	2020/4/7	225/7946
hsa05133	Pertussis	2020/3/7	76/7946
hsa05210	Colorectal cancer	2020/3/7	86/7946
hsa05222	Small cell lung cancer	2020/3/7	92/7946
hsa04657	IL-17 signaling pathway	2020/3/7	94/7946
hsa04010	MAPK signaling pathway	2020/4/7	295/7946
hsa04933	AGE-RAGE signaling pathway in diabetic complications	2020/3/7	100/7946
hsa04668	TNF signaling pathway	2020/3/7	112/7946
hsa05145	Toxoplasmosis	2020/3/7	112/7946
hsa04380	Osteoclast differentiation	2020/3/7	128/7946
hsa04926	Relaxin signaling pathway	2020/3/7	129/7946
hsa05418	Fluid shear stress and atherosclerosis	2020/3/7	139/7946
hsa04932	Non-alcoholic fatty liver disease (NAFLD)	2020/3/7	149/7946
hsa05160	Hepatitis C	2020/3/7	155/7946
hsa05164	Influenza A	2020/3/7	170/7946
hsa04215	Apoptosis - multiple species	2020/2/7	32/7946
hsa05152	Tuberculosis	2020/3/7	180/7946
hsa05016	Huntington disease	2020/3/7	193/7946
hsa05169	Epstein-Barr virus infection	2020/3/7	201/7946
hsa05014	Amyotrophic lateral sclerosis (ALS)	2020/2/7	51/7946
hsa05416	Viral myocarditis	2020/2/7	60/7946
hsa04917	Prolactin signaling pathway	2020/2/7	70/7946
hsa05120	Epithelial cell signaling in Helicobacter pylori infection	2020/2/7	70/7946
hsa04115	p53 signaling pathway	2020/2/7	72/7946
hsa01524	Platinum drug resistance	2020/2/7	73/7946
hsa05212	Pancreatic cancer	2020/2/7	76/7946
hsa05140	Leishmaniasis	2020/2/7	77/7946
hsa05132	Salmonella infection	2020/2/7	80/7946
hsa04662	B cell receptor signaling pathway	2020/2/7	82/7946
hsa05165	Human papillomavirus infection	2020/3/7	330/7946
hsa04211	Longevity regulating pathway	2020/2/7	89/7946
hsa05235	PD-L1 expression and PD-1 checkpoint pathway in cancer	2020/2/7	89/7946
hsa04658	Th1 and Th2 cell differentiation	2020/2/7	92/7946
hsa05323	Rheumatoid arthritis	2020/2/7	93/7946
hsa05142	Chagas disease (American trypanosomiasis)	2020/2/7	102/7946
hsa05146	Amoebiasis	2020/2/7	102/7946
hsa04620	Toll-like receptor signaling pathway	2020/2/7	104/7946
hsa04660	T cell receptor signaling pathway	2020/2/7	104/7946
hsa04659	Th17 cell differentiation	2020/2/7	107/7946
hsa04066	HIF-1 signaling pathway	2020/2/7	109/7946
hsa05135	Yersinia infection	2020/2/7	120/7946
hsa04915	Estrogen signaling pathway	2020/2/7	138/7946
hsa05012	Parkinson disease	2020/2/7	142/7946
hsa05224	Breast cancer	2020/2/7	147/7946
hsa05168	Herpes simplex virus 1 infection	2020/3/7	491/7946
hsa05010	Alzheimer disease	2020/2/7	171/7946
hsa05202	Transcriptional misregulation in cancer	2020/2/7	186/7946
hsa05203	Viral carcinogenesis	2020/2/7	201/7946
hsa05205	Proteoglycans in cancer	2020/2/7	204/7946
hsa04024	cAMP signaling pathway	2020/2/7	216/7946

hsa05166	Human T-cell leukemia virus 1 infection	2020/2/7	219/7946
hsa04014	Ras signaling pathway	2020/2/7	232/7946
hsa05131	Shigellosis	2020/2/7	236/7946
hsa01523	Antifolate resistance	2020/1/7	31/7946
hsa05206	MicroRNAs in cancer	2020/2/7	310/7946

pvalue	p.adjust	qvalue	geneID	Count
1.35E-07	1.39E-05	5.25E-06	2353/836/54205/5970/7422	5
2.76E-06	1.01E-04	3.80E-05	2353/836/54205/5970	4
2.93E-06	1.01E-04	3.80E-05	2353/836/54205/5970	4
5.56E-06	1.43E-04	5.41E-05	2353/836/54205/5970	4
1.20E-05	2.29E-04	8.68E-05	836/54205/5970	3
1.34E-05	2.29E-04	8.68E-05	2353/836/54205/5970	4
1.62E-05	2.38E-04	9.01E-05	2353/836/54205/5970	4
2.05E-05	2.64E-04	9.98E-05	836/54205/5970/7422	4
2.86E-05	3.28E-04	1.24E-04	2353/836/5970	3
4.15E-05	4.28E-04	1.62E-04	2353/836/54205	3
5.08E-05	4.65E-04	1.76E-04	836/54205/5970	3
5.42E-05	4.65E-04	1.76E-04	2353/836/5970	3
5.96E-05	4.72E-04	1.79E-04	2353/836/5970/7422	4
6.53E-05	4.80E-04	1.82E-04	836/5970/7422	3
9.16E-05	5.89E-04	2.23E-04	2353/836/5970	3
9.16E-05	5.89E-04	2.23E-04	836/54205/5970	3
1.36E-04	7.98E-04	3.02E-04	2353/5468/5970	3
1.40E-04	7.98E-04	3.02E-04	2353/5970/7422	3
1.74E-04	9.44E-04	3.57E-04	2353/5970/7422	3
2.14E-04	1.10E-03	4.17E-04	836/54205/5970	3
2.41E-04	1.18E-03	4.46E-04	836/54205/5970	3
3.16E-04	1.46E-03	5.52E-04	836/54205/5970	3
3.26E-04	1.46E-03	5.52E-04	836/54205	2
3.74E-04	1.61E-03	6.07E-04	836/54205/5970	3
4.59E-04	1.89E-03	7.16E-04	836/5468/54205	3
5.18E-04	2.05E-03	7.76E-04	836/54205/5970	3
8.31E-04	3.17E-03	1.20E-03	836/54205	2
1.15E-03	4.23E-03	1.60E-03	836/54205	2
1.56E-03	5.36E-03	2.03E-03	2353/5970	2
1.56E-03	5.36E-03	2.03E-03	836/5970	2
1.65E-03	5.46E-03	2.07E-03	836/54205	2
1.70E-03	5.46E-03	2.07E-03	836/54205	2
1.84E-03	5.71E-03	2.16E-03	5970/7422	2
1.89E-03	5.71E-03	2.16E-03	2353/5970	2
2.03E-03	5.99E-03	2.26E-03	2353/5970	2
2.14E-03	6.11E-03	2.31E-03	2353/5970	2
2.19E-03	6.11E-03	2.31E-03	836/5970/7422	3
2.51E-03	6.63E-03	2.51E-03	5468/5970	2
2.51E-03	6.63E-03	2.51E-03	2353/5970	2
2.68E-03	6.88E-03	2.60E-03	2353/5970	2
2.74E-03	6.88E-03	2.60E-03	2353/7422	2
3.29E-03	7.81E-03	2.95E-03	2353/5970	2
3.29E-03	7.81E-03	2.95E-03	836/5970	2
3.41E-03	7.81E-03	2.95E-03	2353/5970	2
3.41E-03	7.81E-03	2.95E-03	2353/5970	2
3.61E-03	8.08E-03	3.06E-03	2353/5970	2
3.74E-03	8.20E-03	3.10E-03	5970/7422	2
4.52E-03	9.70E-03	3.67E-03	2353/5970	2
5.94E-03	1.25E-02	4.72E-03	2353/5241	2
6.28E-03	1.29E-02	4.89E-03	836/54205	2
6.72E-03	1.35E-02	5.10E-03	2353/5241	2
6.80E-03	1.35E-02	5.10E-03	836/54205/5970	3
9.01E-03	1.75E-02	6.62E-03	836/54205	2
1.06E-02	2.02E-02	7.64E-03	5468/5970	2
1.23E-02	2.30E-02	8.71E-03	836/5970	2
1.27E-02	2.33E-02	8.80E-03	836/7422	2
1.41E-02	2.55E-02	9.64E-03	2353/5970	2

1.45E-02	2.57E-02	9.73E-03	2353/5970	2
1.62E-02	2.82E-02	1.07E-02	5970/7422	2
1.67E-02	2.87E-02	1.08E-02	54205/5970	2
2.70E-02	4.56E-02	1.72E-02	5970	1
2.80E-02	4.65E-02	1.76E-02	836/7422	2
