# Supplementary material for: Determining Pharmacological Mechanisms of Chinese Incompatible Herbs Fuzi and Banxia in Chronic Obstructive Pulmonary Disease: A Systems Pharmacology-Based Study
Source: Evid Based Complement Alternat Med. 2020 Dec 31;2020:8365603. doi: 10.1155/2020/8365603 (PMC7790578; doi:10.1155/2020/8365603)
Supplement: Supplementary Materials — Supplementary Table 1. Gene targets of Fuzi. Supplementary Table 2. Gene targets of Banxia. Supplementary Table 3. Number of targets corresponding to different components. Supplementary Table 4. The data of GO enrichment analysis. Supplementary Table 5. The data of KEGG enrichment analysis. [file 8365603.f1.zip › 8365603.f1/Supplementary_Material_Table_1-5.pdf]

Supplementary Material

1 Supplementary Table

Supplementary Table 1. Gene targets of Fuzi

ID	Name	Target	Symbol
MOL002211	11,14-eicosadienoic acid	Nuclear receptor coactivator 2	NCOA2
MOL002388	Delphin_qt	Prostaglandin G/H synthase 1	PTGS1
MOL002388	Delphin_qt	Prostaglandin G/H synthase 2	PTGS2
MOL002388	Delphin_qt	Nuclear receptor coactivator 2	NCOA2
MOL002392	Deltoin	Prostaglandin G/H synthase 1	PTGS1
MOL002392	Deltoin	Sodium channel protein type 5 subunit alpha	SCN5A
MOL002392	Deltoin	Prostaglandin G/H synthase 2	PTGS2
MOL002392	Deltoin	Acetylcholinesterase	ACHE
MOL002392	Deltoin	Alpha-1B adrenergic receptor	ADRA1B
MOL002392	Deltoin	Beta-2 adrenergic receptor	ADRB2
MOL002392	Deltoin	Trypsin-1	PRSS1
MOL002395	Deoxyandrographolide	Prostaglandin G/H synthase 2	PTGS2
MOL002395	Deoxyandrographolide	Progesterone receptor	PGR

Supplementary Material

MOL002395	Deoxyandrographolide	Nuclear receptor coactivator 2	NCOA2
MOL002395	Deoxyandrographolide	Nuclear receptor coactivator 1	NCOA1
MOL002398	Karanjin	Prostaglandin G/H synthase 1	PTGS1
MOL002398	Karanjin	Estrogen receptor	ESR1
MOL002398	Karanjin	Prostaglandin G/H synthase 2	PTGS2
MOL002398	Karanjin	Serine/threonine-protein kinase Chk1	CHEK1
MOL000359	sitosterol	Progesterone receptor	PGR
MOL000359	sitosterol	Nuclear receptor coactivator 2	NCOA2
MOL000359	sitosterol	Mineralocorticoid receptor	NR3C2

Supplementary Table 2. Gene targets of Banxia

ID	Name	Target	Symbol
MOL000358	beta-sitosterol	Serum paraoxonase/arylesterase 1	PON1
MOL000519	coniferin	Estrogen receptor	ESR1
MOL002714	baicalein	Androgen receptor	AR
MOL000519	coniferin	Androgen receptor	AR

MOL006957	(3S,6S)-3-(benzyl)-6-(4-hydroxybenzyl)piperazine-2,5-quinone	Androgen receptor	AR
MOL002714	baicalein	Cytochrome c	CYCS
MOL002714	baicalein	Vascular endothelial growth factor A	VEGFA
MOL002670	cavidine	Prostaglandin G/H synthase 1	PTGS1
MOL002714	baicalein	Prostaglandin G/H synthase 1	PTGS1
MOL000358	beta-sitosterol	Prostaglandin G/H synthase 1	PTGS1
MOL000449	stigmasterol	Prostaglandin G/H synthase 1	PTGS1
MOL005030	gondoic acid	Prostaglandin G/H synthase 1	PTGS1
MOL006936	10,13-eicosadienoic	Prostaglandin G/H synthase 1	PTGS1
MOL000519	coniferin	Peroxisome proliferator activated receptor gamma	PPARG
MOL002714	baicalein	Apoptosis regulator Bcl-2	BCL2
MOL000358	beta-sitosterol	Apoptosis regulator Bcl-2	BCL2
MOL002670	cavidine	Muscarinic acetylcholine receptor M5	CHRM5
MOL002714	baicalein	Trypsin-1	PRSS1
MOL002670	cavidine	5-hydroxytryptamine receptor 3A	HTR3A

MOL002670	cavidine	Coagulation factor VII	F7
MOL002670	cavidine	Alpha-2C adrenergic receptor	ADRA2C
MOL002670	cavidine	Muscarinic acetylcholine receptor M4	CHRM4
MOL000358	beta-sitosterol	Muscarinic acetylcholine receptor M4	CHRM4
MOL002670	cavidine	Muscarinic acetylcholine receptor M3	CHRM3
MOL000358	beta-sitosterol	Muscarinic acetylcholine receptor M3	CHRM3
MOL000449	stigmasterol	Muscarinic acetylcholine receptor M3	CHRM3
MOL000519	coniferin	Muscarinic acetylcholine receptor M3	CHRM3
MOL000358	beta-sitosterol	Alpha-1A adrenergic receptor	ADRA1A
MOL000449	stigmasterol	Alpha-1A adrenergic receptor	ADRA1A
MOL002714	baicalein	Cellular tumor antigen p53	TP63
MOL000449	stigmasterol	Chymotrypsinogen B	CTRB1
MOL001755	24-ethylcholest-4-en-3-one	Progesterone receptor	PGR
MOL000358	beta-sitosterol	Progesterone receptor	PGR

MOL000449	stigmasterol	Progesterone receptor	PGR
MOL000358	beta-sitosterol	Muscarinic acetylcholine receptor M2	CHRM2
MOL000449	stigmasterol	Muscarinic acetylcholine receptor M2	CHRM2
MOL002714	baicalein	Aryl hydrocarbon receptor	AHR
MOL002714	baicalein	Nuclear receptor coactivator 1	NCOA1
MOL000449	stigmasterol	Nuclear receptor coactivator 1	NCOA1
MOL000519	coniferin	Nuclear receptor coactivator 1	NCOA1
MOL002714	baicalein	Nuclear receptor coactivator 2	NCOA2
MOL000358	beta-sitosterol	Nuclear receptor coactivator 2	NCOA2
MOL000449	stigmasterol	Nuclear receptor coactivator 2	NCOA2
MOL005030	gondoic acid	Nuclear receptor coactivator 2	NCOA2
MOL000519	coniferin	Nuclear receptor coactivator 2	NCOA2
MOL006936	10,13-eicosadienoic	Nuclear receptor coactivator 2	NCOA2
MOL002670	cavidine	cAMP and cAMP-inhibited cGMP 3',5'-cyclic phosphodiesterase 10A	PDE10A
MOL001755	24-ethylcholest-4-en-3-one	Mineralocorticoid receptor	NR3C2

MOL000449	Stigmasterol	Mineralocorticoid receptor	NR3C2
MOL003578	Cycloartenol	Mineralocorticoid receptor	NR3C2
MOL000358	beta-sitosterol	Neuronal acetylcholine receptor subunit alpha-2	CHRNA2
MOL000449	stigmasterol	Aldose reductase	AKR1B1
MOL000358	beta-sitosterol	Gamma-aminobutyric acid receptor subunit alpha-1	GABRA1
MOL000449	stigmasterol	Gamma-aminobutyric acid receptor subunit alpha-1	GABRA1
MOL002670	cavidine	Retinoic acid receptor RXR-beta	RXRB
MOL002714	baicalein	Transcription factor p65	RELA
MOL002714	baicalein	Proto-oncogene c-Fos	FOS
MOL002714	baicalein	Caspase-3	CASP3
MOL002714	baicalein	Hypoxia-inducible factor 1-alpha	HIF1A
MOL002714	baicalein	Fos-related antigen 1	FOSL1
MOL002714	baicalein	G2/mitotic-specific cyclin-B1	CCNB1
MOL002714	baicalein	Insulin-like growth factor II	IGF2
MOL002714	baicalein	NADPH oxidase 5	NOX5

MOL002714	baicalein	Apolipoprotein D	APOD
MOL000358	beta-sitosterol	Caspase-9	CASP9
MOL000358	beta-sitosterol	Caspase-3	CASP3
MOL000358	beta-sitosterol	Caspase-8	CASP8
MOL000358	beta-sitosterol	Protein kinase C alpha type	PRKCA

Supplementary Table 3. Number of targets corresponding to different components

Source	MolName	count
Banxia	baicalein	19
Banxia	beta-sitosterol	15
Banxia	Stigmasterol	11
Banxia	Cavidine	9
Banxia	coniferin	6
Banxia	gondoic acid	2
Banxia	10,13-eicosadienoic	2
Banxia	24-Ethylcholest-4-en-3-one	2
Banxia	(3S,6S)-3-(benzyl)-6-(4-hydroxybenzyl)piperazine-2,5-quinone	1

Supplementary Material

Banxia	Cycloartenol	1
Fuzi	Deltoin	7
Fuzi	Deoxyandrographolide	4
Fuzi	Karanjin	4
Fuzi	Delphin_qt	3
Fuzi	sitosterol	3
Fuzi	11,14-eicosadienoic acid	1

Supplementary Table 4. The data of GO enrichment analysis

批注 [倪1]: Dear editors, to show the tables clearly, we insert the link instead of the tables.

~~ID~~	~~Description~~	~~BgRatio~~	~~pvalue~~	~~p.adjust~~	~~qvalue~~	~~geneID~~	~~Count~~
~~GO:0033613~~	~~activating transcription factor binding~~	~~64/16969~~	~~1.77E-06~~	~~0.00017363~~	~~5.78E-05~~	~~2353/5468/5970~~	~~3~~
~~GO:0046982~~	~~protein heterodimerization activity~~	~~497/16969~~	~~2.37E-05~~	~~0.001162372~~	~~0.000387041~~	~~2353/5468/5970/7422~~	~~4~~
~~GO:0042805~~	~~actinin binding~~	~~29/16969~~	~~5.89E-05~~	~~0.001924371~~	~~0.000640768~~	~~5468/5970~~	~~2~~
~~GO:0004879~~	~~nuclear receptor activity~~	~~46/16969~~	~~0.000149674~~	~~0.002933612~~	~~0.00097682~~	~~5468/5241~~	~~2~~
~~GO:0098531~~	~~transcription factor activity, direct ligand regulated sequence-specific DNA binding~~	~~46/16969~~	~~0.000149674~~	~~0.002933612~~	~~0.00097682~~	~~5468/5241~~	~~2~~
~~GO:0003707~~	~~steroid hormone receptor activity~~	~~54/16969~~	~~0.000206615~~	~~0.003374718~~	~~0.001123698~~	~~5468/5241~~	~~2~~
~~GO:0001228~~	~~DNA-binding transcription activator~~	~~390/16969~~	~~0.000393637~~	~~0.005510923~~	~~0.001835001~~	~~2353/5970/5241~~	~~3~~

	activity, RNA polymerase II-specific						
GO:0000978	RNA polymerase II proximal promoter sequence-specific DNA binding	432/16969	0.000531383	0.005945268	0.001979627	5468/5970/5241	3
GO:0003682	chromatin binding	437/16969	0.000549598	0.005945268	0.001979627	2353/5468/5970	3
GO:0000987	proximal promoter sequence-specific DNA binding	452/16969	0.00060666	0.005945268	0.001979627	5468/5970/5241	3
GO:0005126	cytokine receptor binding	210/16969	0.003072653	0.027374547	0.009115048	836/7422	2
GO:0005172	vascular endothelial growth factor receptor binding	11/16969	0.004529671	0.02971968	0.009895919	7422	1
GO:0016004	phospholipase activator activity	11/16969	0.004529671	0.02971968	0.009895919	836	1
GO:0004861	cyclin-dependent protein serine/threonine kinase inhibitor activity	12/16969	0.004940585	0.02971968	0.009895919	836	1
GO:0004953	icosanoid receptor activity	12/16969	0.004940585	0.02971968	0.009895919	5468	1
GO:0042301	phosphate ion binding	12/16969	0.004940585	0.02971968	0.009895919	5970	1
GO:0060229	lipase activator activity	13/16969	0.005351355	0.02971968	0.009895919	836	1
GO:0005161	platelet-derived growth factor receptor binding	14/16969	0.005761979	0.02971968	0.009895919	7422	1
GO:0036041	long-chain fatty acid binding	14/16969	0.005761979	0.02971968	0.009895919	5468	1
GO:0046965	retinoid X receptor binding	15/16969	0.006172457	0.030245042	0.010070852	5468	1
GO:0005123	death receptor binding	17/16969	0.006992979	0.031171061	0.010379193	836	1

~~GO:0016538~~	~~cyclin-dependent protein serine/threonine kinase regulator activity~~	~~18/16969~~	~~0.007403022~~	~~0.031171061~~	~~0.010379193~~	~~836~~	~~1~~
~~GO:0004190~~	~~aspartic-type endopeptidase activity~~	~~20/16969~~	~~0.008222673~~	~~0.031171061~~	~~0.010379193~~	~~836~~	~~1~~
~~GO:0000979~~	~~RNA polymerase II core promoter sequence-specific DNA binding~~	~~21/16969~~	~~0.00863228~~	~~0.031171061~~	~~0.010379193~~	~~2353~~	~~1~~
~~GO:0001223~~	~~transcription coactivator binding~~	~~21/16969~~	~~0.00863228~~	~~0.031171061~~	~~0.010379193~~	~~5970~~	~~1~~
~~GO:0051393~~	~~alpha-actinin binding~~	~~21/16969~~	~~0.00863228~~	~~0.031171061~~	~~0.010379193~~	~~5468~~	~~1~~
~~GO:0070001~~	~~aspartic-type peptidase activity~~	~~22/16969~~	~~0.009041743~~	~~0.031171061~~	~~0.010379193~~	~~836~~	~~1~~
~~GO:0070412~~	~~R-SMAD binding~~	~~23/16969~~	~~0.009451061~~	~~0.031171061~~	~~0.010379193~~	~~2353~~	~~1~~
~~GO:1901567~~	~~fatty acid derivative binding~~	~~23/16969~~	~~0.009451061~~	~~0.031171061~~	~~0.010379193~~	~~5468~~	~~1~~
~~GO:0001968~~	~~fibronectin binding~~	~~24/16969~~	~~0.009860234~~	~~0.031171061~~	~~0.010379193~~	~~7422~~	~~1~~
~~GO:0042974~~	~~retinoic acid receptor binding~~	~~24/16969~~	~~0.009860234~~	~~0.031171061~~	~~0.010379193~~	~~5468~~	~~1~~
~~GO:0005504~~	~~fatty acid binding~~	~~28/16969~~	~~0.011495477~~	~~0.034311314~~	~~0.01142482~~	~~5468~~	~~1~~
~~GO:0042056~~	~~chemoattractant activity~~	~~29/16969~~	~~0.011903925~~	~~0.034311314~~	~~0.01142482~~	~~7422~~	~~1~~
~~GO:0051059~~	~~NF-kappaB binding~~	~~29/16969~~	~~0.011903925~~	~~0.034311314~~	~~0.01142482~~	~~5970~~	~~1~~
~~GO:0030291~~	~~protein serine/threonine kinase inhibitor activity~~	~~30/16969~~	~~0.01231223~~	~~0.034474243~~	~~0.011479071~~	~~836~~	~~1~~
~~GO:0001046~~	~~core promoter sequence-~~	~~32/16969~~	~~0.013128404~~	~~0.035738433~~	~~0.011900015~~	~~2353~~	~~1~~

	specific DNA binding						
GO:0001102	RNA polymerase II activating transcription factor binding	37/16969	0.015166312	0.040163134	0.013373331	2353	1
GO:0001221	transcription cofactor binding	38/16969	0.01557346	0.040163134	0.013373331	5970	1
GO:0030331	estrogen receptor binding	41/16969	0.016794039	0.041979236	0.013978049	5468	1
GO:0001047	core promoter binding	42/16969	0.017200611	0.041979236	0.013978049	2353	1
GO:0050840	extracellular matrix binding	43/16969	0.017607038	0.041979236	0.013978049	7422	1
GO:0030374	nuclear receptor transcription coactivator activity	45/16969	0.018419461	0.041979236	0.013978049	5468	1
GO:0032813	tumor necrosis factor receptor superfamily binding	45/16969	0.018419461	0.041979236	0.013978049	836	1
GO:0004722	protein serine/threonine phosphatase activity	49/16969	0.020042578	0.043648282	0.014533799	54205	1
GO:0070888	E-box binding	49/16969	0.020042578	0.043648282	0.014533799	5468	1
GO:0004197	cysteine-type endopeptidase activity	56/16969	0.022877499	0.047702019	0.015883594	836	1
GO:0043621	protein self-association	56/16969	0.022877499	0.047702019	0.015883594	5468	1
GO:0033293	monocarboxylic acid binding	59/16969	0.024090311	0.048008818	0.01598575	5468	1
GO:0070491	repressing transcription factor binding	59/16969	0.024090311	0.048008818	0.01598575	5970	1
GO:0004860	protein kinase inhibitor activity	60/16969	0.024494295	0.048008818	0.01598575	836	1

GO..xlsx

Supplementary Table 5. The data of KEGG enrichment analysis

ID	Description	BgRatio	pvalue	p.adjust	qvalue	geneID	Count
hsa04115	p53 signaling pathway	72/8016	7.36E-08	5.42E-06	2.9E-06	BCL2/CASP8/CASP3/CYCS/CASP9/CHEK1/CCNB1	7
hsa04725	Cholinergic synapse	112/8016	8.98E-08	5.42E-06	2.9E-06	BCL2/CHRM3/FOS/ACHE/PRKCA/CHRM2/CHRM4/CHRM5	8
hsa05170	Human immunodeficiency virus 1 infection	212/8016	9.51E-08	5.42E-06	2.9E-06	BCL2/CASP8/RELA/CASP3/CYCS/CASP9/FOS/CHEK1/PRKCA/CCNB1	10
hsa05167	Kaposi sarcoma-associated herpesvirus infection	186/8016	3.7E-07	1.39E-05	7.42E-06	VEGFA/CASP8/PTGS2/RELA/CASP3/HIF1A/CYCS/CASP9/FOS	9
hsa05222	Small cell lung cancer	92/8016	4.05E-07	1.39E-05	7.42E-06	BCL2/PTGS2/RELA/CASP3/CYCS/CASP9/RXRB	7
hsa04215	Apoptosis - multiple species	32/8016	5.6E-07	1.6E-05	8.55E-06	BCL2/CASP8/CASP3/CYCS/CASP9	5
hsa04080	Neuroactive ligand-receptor interaction	340/8016	8.92E-07	2.18E-05	1.17E-05	ADRB2/CHRM3/PRSS1/ADRA1B/CHRM2/ADRA1A/GABRA1/ADRA2C/CHRNA2/CHRM4/CHRM5	11
hsa05161	Hepatitis B	162/8016	1.54E-06	3.29E-05	1.76E-05	BCL2/CASP8/RELA/CASP3/CYCS/CASP9/FOS/PRKCA	8
hsa04919	Thyroid hormone signaling pathway	119/8016	2.34E-06	4.44E-05	2.38E-05	ESR1/HIF1A/CASP9/PRKCA/RXRB/NCOA1/NCOA2	7
hsa04210	Apoptosis	136/8016	5.7E-06	9.74E-05	5.22E-05	BCL2/CASP8/RELA/CASP3/CYCS/CASP9/FOS	7
hsa05162	Measles	138/8016	6.28E-06	9.76E-05	5.23E-05	BCL2/CASP8/RELA/CASP3/CYCS/CASP9/FOS	7

hsa04657	IL-17 signaling pathway	94/8016	8.27E-06	0.000117783	6.31E-05	CASP8/PTGS2/RELA/CASP3/FOS/FOSL1	6
hsa05134	Legionellosis	57/8016	1.06E-05	0.000139183	7.45E-05	CASP8/RELA/CASP3/CYCS/CASP9	5
hsa05163	Human cytomegalovirus infection	225/8016	1.76E-05	0.000215165	0.000115231	VEGFA/CASP8/PTGS2/RELA/CASP3/CYCS/CASP9/PRKCA	8
hsa05145	Toxoplasmosis	112/8016	2.27E-05	0.00025824	0.0001383	BCL2/CASP8/RELA/CASP3/CYCS/CASP9	6
hsa05164	Influenza A	170/8016	2.46E-05	0.000262954	0.000140825	CASP8/RELA/CASP3/PRSS1/CYCS/CASP9/PRKCA	7
hsa01524	Platinum drug resistance	73/8016	3.57E-05	0.000358972	0.000192247	BCL2/CASP8/CASP3/CYCS/CASP9	5
hsa04020	Calcium signaling pathway	193/8016	5.56E-05	0.000527798	0.000282662	ADRB2/CHRM3/PRKCA/ADRA1B/CHRM2/ADRA1A/CHRM5	7
hsa04915	Estrogen signaling pathway	138/8016	7.36E-05	0.000662091	0.000354582	BCL2/ESR1/FOS/PGR/NCOA1/NCOA2	6
hsa05210	Colorectal cancer	86/8016	7.87E-05	0.000672897	0.00036037	BCL2/CASP3/CYCS/CASP9/FOS	5
hsa04970	Salivary secretion	90/8016	9.78E-05	0.00079636	0.00042649	ADRB2/CHRM3/PRKCA/ADRA1B/ADRA1A	5
hsa04261	Adrenergic signaling in cardiomyocytes	149/8016	0.000112616	0.00087533	0.000468783	BCL2/ADRB2/SCN5A/PRKCA/ADRA1B/ADRA1A	6
hsa04933	AGE-RAGE signaling pathway in diabetic complications	100/8016	0.000161202	0.001198501	0.000641857	VEGFA/BCL2/RELA/CASP3/PRKCA	5

~~hsa05014~~	~~Amyotrophic lateral sclerosis (ALS)~~	~~57/8016~~	~~0.000210325~~	~~0.001498564~~	~~0.000802555~~	~~BCL2/CASP3/CYCS/CASP9~~	~~4~~
~~hsa04659~~	~~Th17 cell differentiation~~	~~107/8016~~	~~0.000221566~~	~~0.001515512~~	~~0.000811632~~	~~RELA/HIF1A/FOS/AHR/RXRB~~	~~5~~
~~hsa04370~~	~~VEGF signaling pathway~~	~~59/8016~~	~~0.000240504~~	~~0.001530274~~	~~0.000819537~~	~~VEGFA/PTGS2/CASP9/PRKCA~~	~~4~~
~~hsa04066~~	~~HIF-1 signaling pathway~~	~~109/8016~~	~~0.000241622~~	~~0.001530274~~	~~0.000819537~~	~~VEGFA/BCL2/RELA/HIF1A/PRKCA~~	~~5~~
~~hsa05416~~	~~Viral myocarditis~~	~~60/8016~~	~~0.000256709~~	~~0.001567756~~	~~0.000839611~~	~~CASP8/CASP3/CYCS/CASP9~~	~~4~~
~~hsa04668~~	~~TNF signaling pathway~~	~~112/8016~~	~~0.000274266~~	~~0.001617227~~	~~0.000866105~~	~~CASP8/PTGS2/RELA/CASP3/FOS~~	~~5~~
~~hsa04726~~	~~Serotonergic synapse~~	~~115/8016~~	~~0.000310165~~	~~0.001743051~~	~~0.00093349~~	~~PTGS2/CASP3/PTGS1/HTR3A/PRKCA~~	~~5~~
~~hsa05152~~	~~Tuberculosis~~	~~180/8016~~	~~0.000315992~~	~~0.001743051~~	~~0.00093349~~	~~BCL2/CASP8/RELA/CASP3/CYCS/CASP9~~	~~6~~
~~hsa05169~~	~~Epstein-Barr virus infection~~	~~201/8016~~	~~0.00056982~~	~~0.003031324~~	~~0.001623424~~	~~BCL2/CASP8/RELA/CASP3/CYCS/CASP9~~	~~6~~
~~hsa05130~~	~~Pathogenic Escherichia coli infection~~	~~202/8016~~	~~0.000584992~~	~~0.003031324~~	~~0.001623424~~	~~CASP8/RELA/CASP3/CYCS/CASP9/FOS~~	~~6~~
~~hsa05205~~	~~Proteoglycans in cancer~~	~~204/8016~~	~~0.000616282~~	~~0.003099535~~	~~0.001659954~~	~~VEGFA/ESR1/IGF2/CASP3/HIF1A/PRKCA~~	~~6~~
~~hsa05132~~	~~Salmonella infection~~	~~214/8016~~	~~0.000792812~~	~~0.003873454~~	~~0.002074426~~	~~BCL2/CASP8/RELA/CASP3/CYCS/FOS~~	~~6~~
~~hsa05160~~	~~Hepatitis C~~	~~155/8016~~	~~0.001210174~~	~~0.005748327~~	~~0.003078513~~	~~CASP8/RELA/CASP3/CYCS/CASP9~~	~~5~~

hsa05215	Prostate cancer	97/8016	0.00158571	0.007328553	0.003924802	BCL2/RELA/AR/CASP9	4
hsa04151	PI3K-Akt signaling pathway	354/8016	0.002187139	0.0096352	0.005160126	VEGFA/BCL2/IGF2/RELA/CASP9/PRKCA/CHRM2	7
hsa04928	Parathyroid hormone synthesis, secretion and action	106/8016	0.002197502	0.0096352	0.005160126	BCL2/FOS/PRKCA/RXRB	4
hsa04923	Regulation of lipolysis in adipocytes	55/8016	0.00290397	0.01241447	0.006648562	ADRB2/PTGS2/PTGS1	3
hsa04010	MAPK signaling pathway	295/8016	0.004052689	0.01690268	0.00905222	VEGFA/IGF2/RELA/CASP3/FOS/PRKCA	6
hsa04380	Osteoclast differentiation	128/8016	0.004343165	0.017682885	0.009470058	PPARG/RELA/FOS/FOSL1	4
hsa04926	Relaxin signaling pathway	129/8016	0.004465281	0.01775728	0.009509901	VEGFA/RELA/FOS/PRKCA	4
hsa05223	Non-small cell lung cancer	66/8016	0.004867202	0.018915717	0.010130301	CASP9/PRKCA/RXRB	3
hsa05206	MicroRNAs in cancer	310/8016	0.005161881	0.019615148	0.010504881	VEGFA/BCL2/TP63/PTGS2/CASP3/PRKCA	6
hsa04917	Prolactin signaling pathway	70/8016	0.005738635	0.021162244	0.011333427	ESR1/RELA/FOS	3
hsa05418	Fluid shear stress and atherosclerosis	139/8016	0.005816523	0.021162244	0.011333427	VEGFA/BCL2/RELA/FOS	4

hsa05224	Breast cancer	147/8016	0.0070766 68	0.0245874 94	0.0131678 18	ESR1/FOS/PGR/NCOA1	4
hsa05133	Pertussis	76/8016	0.0072116 17	0.0245874 94	0.0131678 18	RELA/CASP3/FOS	3
hsa05212	Pancreatic cancer	76/8016	0.0072116 17	0.0245874 94	0.0131678 18	VEGFA/RELA/CASP9	3
hsa04932	Non-alcoholic fatty liver disease (NAFLD)	149/8016	0.0074178 36	0.0245874 94	0.0131678 18	CASP8/RELA/CASP3/CYCS	4
hsa05140	Leishmaniasis	77/8016	0.0074768 99	0.0245874 94	0.0131678 18	PTGS2/RELA/FOS	3
hsa01521	EGFR tyrosine kinase inhibitor resistance	79/8016	0.0080247 01	0.0258910 15	0.0138659 17	VEGFA/BCL2/PRKCA	3
hsa04742	Taste transduction	83/8016	0.0091900 44	0.0291018 04	0.0155854 54	CHRM3/HTR3A/GABRA1	3
hsa04022	cGMP-PKG signaling pathway	167/8016	0.0109879 08	0.0339418 08	0.0181775 15	ADRB2/ADRA1B/ADRA1A/ADRA2C	4
hsa05235	PD-L1 expression and PD-1 checkpoint pathway in cancer	89/8016	0.0111154 46	0.0339418 08	0.0181775 15	RELA/HIF1A/FOS	3
hsa05016	Huntington disease	264/8016	0.0117237 36	0.0351111 97	0.0188037 81	PPARG/CASP8/CASP3/CYCS/CASP9	5
hsa05010	Alzheimer disease	171/8016	0.0119090 61	0.0351111 97	0.0188037 81	CASP8/CASP3/CYCS/CASP9	4

hsa01522	Endocrine resistance	98/8016	0.014411657	0.0410732 22	0.0219967 39	BCL2/ESR1/FOS	3
hsa05231	Choline metabolism in cancer	98/8016	0.0144116 57	0.0410732 22	0.0219967 39	HIF1A/FOS/PRKCA	3
hsa04960	Aldosterone-regulated sodium reabsorption	37/8016	0.01589521	0.0415492 79	0.0222516 91	NR3C2/PRKCA	2
hsa05216	Thyroid cancer	37/8016	0.01589521	0.0415492 79	0.0222516 91	PPARG/RXRB	2
hsa04064	NF-kappa B signaling pathway	102/8016	0.0160365 64	0.0415492 79	0.0222516 91	BCL2/PTGS2/RELA	3
hsa04972	Pancreatic secretion	102/8016	0.0160365 64	0.0415492 79	0.0222516 91	CHRM3/PRSS1/PRKCA	3
hsa05142	Chagas disease (American trypanosomiasis)	102/8016	0.0160365 64	0.0415492 79	0.0222516 91	CASP8/RELA/FOS	3
hsa05146	Amoebiasis	102/8016	0.0160365 64	0.0415492 79	0.0222516 91	RELA/CASP3/PRKCA	3
hsa04620	Toll-like receptor signaling pathway	104/8016	0.0168863 05	0.04246409	0.0227416 18	CASP8/RELA/FOS	3
hsa04625	C-type lectin receptor signaling pathway	104/8016	0.0168863 05	0.04246409	0.0227416 18	CASP8/PTGS2/RELA	3

KEGG..xlsx
